# Supplementary material for: Genome-wide identification of neuronal activity-regulated genes in Drosophila
Source: eLife. 2016 Dec 9;5:e19942. doi: 10.7554/eLife.19942 (PMC5148613; doi:10.7554/eLife.19942)
Supplement: Figure 1—source data 1. — DOI: http://dx.doi.org/10.7554/eLife.19942.003 [file elife-19942-fig1-data1.docx]

**Figure 1 – Source Data 1. *ChR2-XXL-*induced ARGs in fly brains.**

| ranking | gene_id (ChR2-XXL-induced) | log2 fold change at 60min | Light induced? |
| --- | --- | --- | --- |
| 1 | Hr38 | 6.49 |  |
| 2 | sr | 3.37 |  |
| 3 | CG14186 | 2.74 |  |
| 4 | CG7995 | 2.06 |  |
| 5 | CG30497 | 1.66 |  |
| 6 | CG7218 | 1.27 |  |
| 7 | CG13055 | 1.25 | induced by light |
| 8 | CG15745 | 1.21 |  |
| 9 | CG13999 | 1.10 |  |
| 10 | cbt | 1.07 |  |
| 11 | l(1)G0148 | 1.04 |  |
| 12 | CG42856 | 0.96 |  |
| 13 | CG17778 | 0.94 |  |
| 14 | CG8910 | 0.93 |  |
| 15 | CG14024 | 0.87 |  |
| 16 | Hsromega | 0.83 | induced by light |
| 17 | Dref | 0.81 |  |
| 18 | CG11221 | 0.77 |  |
| 19 | Hex-A | 0.67 |  |
| 20 | Atf-2 | 0.64 |  |
| 21 | skl | 0.63 |  |
| 22 | CG6201 | 0.62 |  |
| 23 | Ilp3 | 0.60 |  |
| 24 | Pk61C | 0.59 |  |
| 25 | CG11293 | 0.59 |  |
| 26 | CG17734 | 0.58 |  |
| 27 | aay | 0.56 |  |
| 28 | CG11597 | 0.56 |  |
| 29 | ort | 0.55 |  |
| 30 | cwo | 0.55 |  |
| 31 | CG42708 | 0.55 |  |
| 32 | CG10960 | 0.54 |  |
| 33 | CG8321 | 0.53 |  |
| 34 | slim | 0.53 |  |
| 35 | CG6231 | 0.52 |  |
| 36 | CG1607 | 0.52 |  |
| 37 | sty | 0.52 |  |
| 38 | CG42261 | 0.51 |  |
| 39 | CG6051 | 0.51 |  |
| 40 | Mctp | 0.47 |  |
| 41 | Eip75B | 0.45 |  |
| 42 | Hop | 0.45 |  |
| 43 | CG13868 | 0.45 |  |
| 44 | CG34155 | 0.44 |  |
| 45 | Cyp4ac3 | 0.44 |  |
| 46 | loco | 0.44 |  |
| 47 | jeb | 0.43 |  |
| 48 | Mkp3 | 0.43 |  |
| 49 | CG9413 | 0.43 |  |
| 50 | CG5071 | 0.42 |  |
| 51 | CG8177 | 0.41 |  |
| 52 | Gap1 | 0.40 |  |
| 53 | Lsd-2 | 0.40 |  |
| 54 | CG4577 | 0.40 |  |
| 55 | cv-c | 0.40 |  |
| 56 | CG6006 | 0.40 |  |
| 57 | raw | 0.40 |  |
| 58 | CG16717 | 0.39 |  |
| 59 | CG3961 | 0.39 |  |
| 60 | BRWD3 | 0.39 |  |
| 61 | Pect | 0.39 |  |
| 62 | CG9005 | 0.39 |  |
| 63 | CG4797 | 0.37 |  |
| 64 | Rep | 0.37 |  |
| 65 | Su(z)2 | 0.37 |  |
| 66 | Hsp23 | 0.37 | induced by light |
| 67 | Drep-1 | 0.37 |  |
| 68 | Atox1 | 0.36 |  |
| 69 | CG9328 | 0.36 |  |
| 70 | uzip | 0.36 |  |
| 71 | Hr78 | 0.36 |  |
| 72 | qkr58E-3 | 0.36 |  |
| 73 | puc | 0.36 |  |
| 74 | CG4404 | 0.35 |  |
| 75 | Flo | 0.34 |  |
| 76 | CG7510 | 0.34 |  |
| 77 | CG7378 | 0.33 |  |
| 78 | Rgk3 | 0.32 |  |
| 79 | CG31808 | 0.32 |  |
| 80 | CG1316 | 0.31 |  |
| 81 | tipE | 0.30 |  |
| 82 | Sln | 0.30 |  |
| 83 | CG18812 | 0.30 |  |
| 84 | CG8861 | 0.30 |  |
| 85 | CG5830 | 0.30 |  |
| 86 | vvl | 0.30 |  |
| 87 | CG8026 | 0.29 |  |
| 88 | glec | 0.29 |  |
| 89 | CG42709 | 0.29 |  |
| 90 | Ubc-E2H | 0.29 |  |
| 91 | phyl | 0.29 |  |
| 92 | Ubi-p63E | 0.28 |  |
| 93 | sra | 0.28 |  |
| 94 | Rph | 0.28 |  |
| 95 | qtc | 0.28 |  |
| 96 | CG9318 | 0.28 |  |
