# Supplementary material for: Genome-wide identification of neuronal activity-regulated genes in Drosophila
Source: eLife. 2016 Dec 9;5:e19942. doi: 10.7554/eLife.19942 (PMC5148613; doi:10.7554/eLife.19942)
Supplement: Figure 3—source data 1. — DOI: http://dx.doi.org/10.7554/eLife.19942.008 [file elife-19942-fig3-data1.docx]

**Figure 3 – Source Data 1. *dTrpA1-*induced ARGs in fly brains.**

| ranking | gene_id (*Elav-dTrpA1*) | log2 Fold Changes at 60 min |
| --- | --- | --- |
| 1 | Hr38 | 5.23 |
| 2 | tld | 4.49 |
| 3 | CG3288 | 3.21 |
| 4 | CG30497 | 2.54 |
| 5 | CG13055 | 2.38 |
| 6 | CG7995 | 2.27 |
| 7 | CG13054 | 2.12 |
| 8 | sr | 2.08 |
| 9 | CG14186 | 1.97 |
| 10 | grass | 1.81 |
| 11 | CG15745 | 1.55 |
| 12 | CG17778 | 1.38 |
| 13 | l(1)G0148 | 1.31 |
| 14 | CG10514 | 1.20 |
| 15 | CG11221 | 1.14 |
| 16 | ntc | 1.11 |
| 17 | Inos | 0.83 |
| 18 | CG8910 | 0.82 |
| 19 | ssp | 0.81 |
| 20 | Rhp | 0.78 |
| 21 | CG14024 | 0.76 |
| 22 | CG7218 | 0.75 |
| 23 | CG13868 | 0.73 |
| 24 | CG13255 | 0.71 |
| 25 | CG17734 | 0.70 |
| 26 | shn | 0.69 |
| 27 | CG42708 | 0.65 |
| 28 | CG13999 | 0.64 |
| 29 | Gap1 | 0.64 |
| 30 | CG10960 | 0.64 |
| 31 | Bzd | 0.63 |
| 32 | Dgk | 0.63 |
| 33 | fat-spondin | 0.62 |
| 34 | CG12290 | 0.62 |
| 35 | Teh4 | 0.62 |
| 36 | Eip75B | 0.61 |
| 37 | Rpn6 | 0.58 |
| 38 | Srp14 | 0.58 |
| 39 | CG42856 | 0.58 |
| 40 | CG5537 | 0.58 |
| 41 | cv-c | 0.57 |
| 42 | Act42A | 0.57 |
| 43 | mrt | 0.56 |
| 44 | CG5357 | 0.55 |
| 45 | CG33229 | 0.53 |
| 46 | CG34372 | 0.51 |
| 47 | CG42796 | 0.50 |
| 48 | CG12821 | 0.50 |
| 49 | CG4080 | 0.50 |
| 50 | capa | 0.49 |
| 51 | CG6051 | 0.48 |
| 52 | Gga | 0.48 |
| 53 | m-cup | 0.48 |
| 54 | CG34294 | 0.48 |
| 55 | Fmrf | 0.47 |
| 56 | Hr78 | 0.47 |
| 57 | Got2 | 0.47 |
| 58 | Dak1 | 0.47 |
| 59 | CG11000 | 0.46 |
| 60 | baf | 0.44 |
| 61 | CG14082 | 0.44 |
| 62 | CG9743 | 0.42 |
| 63 | Atf-2 | 0.41 |
| 64 | mib1 | 0.41 |
| 65 | Jra | 0.41 |
| 66 | JhI-21 | 0.40 |
| 67 | haf | 0.40 |
| 68 | CG32032 | 0.40 |
| 69 | qtc | 0.40 |
| 70 | BRWD3 | 0.39 |
| 71 | CG8177 | 0.39 |
| 72 | CG6201 | 0.38 |
| 73 | CG15803 | 0.38 |
| 74 | ksr | 0.38 |
| 75 | CG1607 | 0.38 |
| 76 | Rph | 0.37 |
| 77 | CG1943 | 0.37 |
| 78 | CG7510 | 0.36 |
| 79 | bnb | 0.36 |
| 80 | Teh2 | 0.36 |
| 81 | MP1 | 0.35 |
| 82 | CG7991 | 0.34 |
| 83 | Rab6 | 0.34 |
| 84 | svr | 0.34 |
| 85 | BM-40-SPARC | 0.33 |
| 86 | Npc2a | 0.33 |
| 87 | alphaTub84B | 0.33 |
| 88 | sty | 0.31 |
| 89 | CG6282 | 0.31 |
| 90 | Mctp | 0.30 |
| 91 | sda | 0.30 |
| 92 | raw | 0.30 |
| 93 | CG13248 | 0.30 |
| 94 | spir | 0.29 |
| 95 | Aats-thr | 0.29 |
| 96 | Syn1 | 0.28 |
| 97 | Neu3 | 0.28 |
