# Supplementary material for: Genome-wide identification of neuronal activity-regulated genes in Drosophila
Source: eLife. 2016 Dec 9;5:e19942. doi: 10.7554/eLife.19942 (PMC5148613; doi:10.7554/eLife.19942)
Supplement: Figure 3—source data 2. — DOI: http://dx.doi.org/10.7554/eLife.19942.009 [file elife-19942-fig3-data2.docx]

**Figure 3 – Source Data 2. KCl-induced ARGs in 60 min in fly brains.**

| ranking | gene_id (KCl 60') | log2 Fold Changes at 60 min |
| --- | --- | --- |
| 1 | Hsp26 | 4.42 |
| 2 | Hsp27 | 4.28 |
| 3 | Hsp70Bc | 4.05 |
| 4 | Hsp68 | 3.54 |
| 5 | alphagamma-element:CR32865 | 3.48 |
| 6 | Hsp23 | 2.24 |
| 7 | DnaJ-1 | 1.62 |
| 8 | Hsp83 | 1.60 |
| 9 | stv | 1.41 |
| 10 | CG8620 | 1.33 |
| 11 | Hr38 | 1.32 |
| 12 | CG14186 | 1.22 |
| 13 | CG13055 | 0.98 |
| 14 | Arc1 | 0.95 |
| 15 | CG17778 | 0.79 |
| 16 | CG30497 | 0.76 |
| 17 | Hsromega | 0.76 |
| 18 | Hsp67Bc | 0.62 |
| 19 | Xrp1 | 0.59 |
| 20 | sr | 0.56 |
| 21 | Ubi-p63E | 0.55 |
| 22 | Kdm4B | 0.54 |
| 23 | CG4577 | 0.54 |
| 24 | Hop | 0.53 |
| 25 | hop | 0.53 |
| 26 | CG12641 | 0.52 |
| 27 | Jra | 0.51 |
| 28 | CG5060 | 0.49 |
| 29 | CG14024 | 0.49 |
| 30 | neuroligin | 0.46 |
| 31 | Dscam3 | 0.45 |
| 32 | CG10543 | 0.45 |
| 33 | CG12734 | 0.45 |
| 34 | CG42750 | 0.44 |
| 35 | npf | 0.44 |
| 36 | ct | 0.43 |
| 37 | Nos | 0.43 |
| 38 | Mctp | 0.43 |
