# Supplementary material for: Genome-wide identification of neuronal activity-regulated genes in Drosophila
Source: eLife. 2016 Dec 9;5:e19942. doi: 10.7554/eLife.19942 (PMC5148613; doi:10.7554/eLife.19942)
Supplement: Figure 3—source data 3. — DOI: http://dx.doi.org/10.7554/eLife.19942.010 [file elife-19942-fig3-data3.docx]

**Figure 3 – Source Data 3. KCl*-*induced ARGs in 90 min in fly brains.**

| ranking | gene_id (KCl 90') | log2 Fold Changes at 90 min |
| --- | --- | --- |
| 1 | Hsp26 | 4.73 |
| 2 | Hsp70Bc | 4.53 |
| 3 | Hsp27 | 4.52 |
| 4 | Hsp68 | 3.66 |
| 5 | Hsp23 | 2.55 |
| 6 | Hr38 | 2.38 |
| 7 | snmRNA:331 | 1.84 |
| 8 | DnaJ-1 | 1.71 |
| 9 | Hsp83 | 1.66 |
| 10 | CG14186 | 1.54 |
| 11 | stv | 1.39 |
| 12 | CG30497 | 1.24 |
| 13 | Hsp67Bc | 1.08 |
| 14 | sr | 1.05 |
| 15 | Ubi-p63E | 0.89 |
| 16 | CG17778 | 0.84 |
| 17 | CG10383 | 0.84 |
| 18 | Hop | 0.82 |
| 19 | Jra | 0.78 |
| 20 | CG13055 | 0.74 |
| 21 | CG14024 | 0.73 |
| 22 | CG13322 | 0.65 |
| 23 | ZC3H3 | 0.58 |
| 24 | Frq2 | 0.57 |
| 25 | Mctp | 0.56 |
| 26 | Dref | 0.56 |
| 27 | mei-P26 | 0.56 |
| 28 | nAcRalpha-30D | 0.55 |
| 29 | Kdm4B | 0.55 |
| 30 | Camta | 0.54 |
| 31 | Hsromega | 0.52 |
| 32 | nmo | 0.52 |
| 33 | Syx1A | 0.52 |
| 34 | brp | 0.52 |
| 35 | CG34362 | 0.52 |
| 36 | Rdl | 0.51 |
| 37 | CG8910 | 0.51 |
| 38 | CR30068 | 0.51 |
| 39 | CG6619 | 0.51 |
| 40 | Sh | 0.51 |
| 41 | CG8301 | 0.51 |
| 42 | CG13954 | 0.50 |
| 43 | CG3409 | 0.50 |
| 44 | Vmat | 0.50 |
| 45 | Acf1 | 0.50 |
| 46 | CG33639 | 0.49 |
| 47 | Wnk | 0.49 |
| 48 | scrt | 0.49 |
| 49 | CG4577 | 0.49 |
| 50 | bol | 0.49 |
| 51 | kcc | 0.49 |
| 52 | CG4294 | 0.49 |
| 53 | 4EHP | 0.48 |
| 54 | Su(z)2 | 0.48 |
| 55 | ct | 0.48 |
| 56 | CG42261 | 0.48 |
| 57 | Mad | 0.47 |
| 58 | l(1)G0148 | 0.47 |
| 59 | Sln | 0.47 |
| 60 | CG31191 | 0.47 |
| 61 | Ih | 0.47 |
| 62 | nAcRalpha-34E | 0.47 |
| 63 | CG4893 | 0.47 |
| 64 | gpp | 0.47 |
| 65 | CG5890 | 0.47 |
| 66 | ari-1 | 0.47 |
| 67 | fred | 0.46 |
| 68 | CG42797 | 0.46 |
| 69 | CG17378 | 0.46 |
| 70 | stau | 0.46 |
| 71 | Mnn1 | 0.46 |
| 72 | CG42404 | 0.46 |
| 73 | CG42708 | 0.46 |
| 74 | CG17193 | 0.45 |
| 75 | Thd1 | 0.45 |
| 76 | CG13739 | 0.45 |
| 77 | Cha | 0.45 |
| 78 | CG9328 | 0.45 |
| 79 | CG12734 | 0.45 |
| 80 | CG13928 | 0.45 |
| 81 | Syt1 | 0.44 |
| 82 | Xrp1 | 0.44 |
| 83 | ple | 0.44 |
| 84 | RSG7 | 0.43 |
| 85 | CG1695 | 0.43 |
| 86 | CG30116 | 0.43 |
| 87 | CG31772 | 0.43 |
| 88 | CG15772 | 0.43 |
| 89 | Csp | 0.43 |
| 90 | CG42784 | 0.43 |
| 91 | PIP5K59B | 0.43 |
| 92 | CG34353 | 0.43 |
| 93 | fd85E | 0.43 |
| 94 | neuroligin | 0.42 |
| 95 | fru | 0.42 |
| 96 | Nhe2 | 0.42 |
| 97 | cpx | 0.42 |
| 98 | heph | 0.41 |
| 99 | shi | 0.41 |
| 100 | DopEcR | 0.41 |
| 101 | Hsc70-4 | 0.41 |
| 102 | 5-HT1A | 0.40 |
| 103 | CG13868 | 0.40 |
| 104 | Hsf | 0.40 |
| 105 | phyl | 0.40 |
| 106 | tipE | 0.40 |
| 107 | PNUTS | 0.37 |
