# Supplementary material for: Genome-wide identification of neuronal activity-regulated genes in Drosophila
Source: eLife. 2016 Dec 9;5:e19942. doi: 10.7554/eLife.19942 (PMC5148613; doi:10.7554/eLife.19942)
Supplement: Figure 3—source data 4. — DOI: http://dx.doi.org/10.7554/eLife.19942.011 [file elife-19942-fig3-data4.docx]

**Figure 3 – Source Data 4. Overlapped ARGs in brains.**

| *ChR2-XXL*/*dTrpA1* only | *dTrpA1*/KCl only | *ChR2-XXL*/KCl only | All |
| --- | --- | --- | --- |
| CG7995 | Jra | Hsromega | Hr38 |
| CG7218 |  | Dref | sr |
| CG15745 |  | CG42261 | CG14186 |
| CG13999 |  | Hop | CG30497 |
| CG42856 |  | CG4577 | CG13055 |
| CG11221 |  | Su(z)2 | l(1)G0148 |
| Atf-2 |  | Hsp23 | CG17778 |
| CG6201 |  | CG9328 | CG8910 |
| CG17734 |  | tipE | CG14024 |
| CG10960 |  | Sln | CG42708 |
| CG1607 |  | phyl | Mctp |
| sty |  | Ubi-p63E | CG13868 |
| CG6051 |  |  |  |
| Eip75B |  |  |  |
| CG8177 |  |  |  |
| Gap1 |  |  |  |
| cv-c |  |  |  |
| raw |  |  |  |
| BRWD3 |  |  |  |
| Hr78 |  |  |  |
| CG7510 |  |  |  |
| Rph |  |  |  |
| qtc |  |  |  |
