# Supplementary material for: Genome-wide identification of neuronal activity-regulated genes in Drosophila
Source: eLife. 2016 Dec 9;5:e19942. doi: 10.7554/eLife.19942 (PMC5148613; doi:10.7554/eLife.19942)
Supplement: Figure 5—source data 1. — DOI: http://dx.doi.org/10.7554/eLife.19942.020 [file elife-19942-fig5-data1.docx]

**Figure 5 – Source Data 1. ARGs induced in DA neurons.**

| Ranking | gene_id | log2 Fold Changes at 60 min |
| --- | --- | --- |
| 1 | Hr38 | 4.32 |
| 2 | CG5618 | 4.08 |
| 3 | Mtk | 4.08 |
| 4 | CG14186 | 2.22 |
| 5 | CG13012 | 2.15 |
| 6 | Fmrf | 2.12 |
| 7 | CG13054 | 1.87 |
| 8 | Drs | 1.86 |
| 9 | CG34136 | 1.76 |
| 10 | sr | 1.65 |
| 11 | CG13055 | 1.53 |
| 12 | CrebA | 1.46 |
| 13 | l(1)G0148 | 1.33 |
| 14 | GstE1 | 1.33 |
| 15 | cbt | 1.32 |
| 16 | CG42807 | 1.31 |
| 17 | grass | 1.26 |
| 18 | cv-c | 1.18 |
| 19 | CG14629 | 1.17 |
| 20 | GstD1 | 1.16 |
| 21 | Idgf2 | 1.11 |
| 22 | CG1572 | 1.11 |
| 23 | CG14966 | 1.10 |
| 24 | CG8407 | 1.10 |
| 25 | l(2)efl | 1.09 |
| 26 | Idgf1 | 1.07 |
| 27 | Inos | 1.03 |
| 28 | CG4288 | 1.03 |
| 29 | Hsc70-3 | 1.02 |
| 30 | CG14274 | 1.02 |
| 31 | CG32276 | 1.01 |
| 32 | CG3847 | 0.99 |
| 33 | bip1 | 0.98 |
| 34 | Tsp42Ea | 0.98 |
| 35 | CG6310 | 0.97 |
| 36 | GstE6 | 0.97 |
| 37 | Tret1-1 | 0.96 |
| 38 | Cypl | 0.94 |
| 39 | aay | 0.93 |
| 40 | Pdk | 0.92 |
| 41 | lectin-28C | 0.91 |
| 42 | Gp93 | 0.90 |
| 43 | CG15877 | 0.90 |
| 44 | SmD1 | 0.90 |
| 45 | Act42A | 0.89 |
| 46 | CG6041 | 0.88 |
| 47 | PDCD-5 | 0.84 |
| 48 | CG17778 | 0.83 |
| 49 | ced-6 | 0.83 |
| 50 | dm | 0.81 |
| 51 | CG34331 | 0.81 |
| 52 | CG9705 | 0.80 |
| 53 | CG31457 | 0.80 |
| 54 | D19B | 0.79 |
| 55 | ntc | 0.79 |
| 56 | kar | 0.79 |
| 57 | Tapdelta | 0.78 |
| 58 | CG7970 | 0.77 |
| 59 | CG7911 | 0.77 |
| 60 | CG11221 | 0.77 |
| 61 | Ubi-p63E | 0.76 |
| 62 | Ssl1 | 0.75 |
| 63 | ran | 0.75 |
| 64 | CG8066 | 0.74 |
| 65 | icln | 0.73 |
| 66 | CG14463 | 0.72 |
| 67 | Tfb1 | 0.72 |
| 68 | CG12338 | 0.72 |
| 69 | CG8635 | 0.70 |
| 70 | CG17734 | 0.69 |
| 71 | Ance-5 | 0.68 |
| 72 | CG13895 | 0.68 |
| 73 | CG13868 | 0.67 |
| 74 | CG6199 | 0.67 |
| 75 | CG5792 | 0.65 |
| 76 | AnnIX | 0.64 |
| 77 | ref(2)P | 0.63 |
| 78 | CG7946 | 0.63 |
| 79 | Eip71CD | 0.63 |
| 80 | CG10863 | 0.61 |
| 81 | CG6206 | 0.61 |
| 82 | baf | 0.60 |
| 83 | koko | 0.60 |
| 84 | Sec61beta | 0.59 |
| 85 | maf-S | 0.56 |
