# Supplementary material for: Genome-wide identification of neuronal activity-regulated genes in Drosophila
Source: eLife. 2016 Dec 9;5:e19942. doi: 10.7554/eLife.19942 (PMC5148613; doi:10.7554/eLife.19942)
Supplement: Figure 5—source data 2. — DOI: http://dx.doi.org/10.7554/eLife.19942.021 [file elife-19942-fig5-data2.docx]

**Figure 5 – Source Data 2. ARGs induced in PDF+ neurons.**

| Ranking | gene_id (PDF-dTrpA1) | log2 Fold Changes at 60 min |
| --- | --- | --- |
| 1 | CG9313 | 3.37 |
| 2 | l(2)efl | 2.57 |
| 3 | bnb | 1.92 |
| 4 | JhI-21 | 1.91 |
| 5 | vir-1 | 1.82 |
| 6 | DAT | 1.77 |
| 7 | Tdc2 | 1.69 |
| 8 | cv-2 | 1.67 |
| 9 | CG8128 | 1.65 |
| 10 | CG11221 | 1.61 |
| 11 | Obp44a | 1.54 |
| 12 | CG17778 | 1.40 |
| 13 | CG14186 | 1.35 |
| 14 | CG13707 | 1.32 |
| 15 | stv | 1.32 |
| 16 | CG15209 | 1.29 |
| 17 | CG16947 | 1.27 |
| 18 | CG13631 | 1.21 |
| 19 | CG9686 | 1.18 |
| 20 | Tsf1 | 1.18 |
| 21 | CG16857 | 1.14 |
| 22 | Msr-110 | 1.12 |
| 23 | Irc | 1.08 |
| 24 | CG30497 | 1.08 |
| 25 | CG14141 | 1.07 |
| 26 | CG3308 | 1.06 |
| 27 | CG7607 | 1.06 |
| 28 | Ugt35b | 1.04 |
| 29 | Ubqn | 1.03 |
| 30 | CG7272 | 1.01 |
| 31 | Csas | 1.01 |
| 32 | CG16936 | 1.01 |
| 33 | mfas | 1.00 |
| 34 | Jheh2 | 0.97 |
| 35 | l(1)G0148 | 0.97 |
| 36 | Cct5 | 0.97 |
| 37 | CG9360 | 0.96 |
| 38 | Cyp28d1 | 0.96 |
| 39 | Pdk | 0.95 |
| 40 | CG13255 | 0.95 |
| 41 | CG4857 | 0.93 |
| 42 | nrv2 | 0.91 |
| 43 | CG31706 | 0.90 |
| 44 | CG5254 | 0.90 |
| 45 | CG7888 | 0.90 |
| 46 | cbt | 0.89 |
| 47 | CG3940 | 0.89 |
| 48 | Spn43Ab | 0.89 |
| 49 | CG3104 | 0.88 |
| 50 | CG1545 | 0.84 |
| 51 | Tcp-1eta | 0.83 |
| 52 | Act42A | 0.82 |
| 53 | CG12269 | 0.82 |
| 54 | CG5895 | 0.82 |
| 55 | pcs | 0.81 |
| 56 | CG7033 | 0.81 |
| 57 | MtnA | 0.80 |
| 58 | Oatp58Dc | 0.79 |
| 59 | CG4409 | 0.79 |
| 60 | CG32521 | 0.79 |
| 61 | Irk2 | 0.76 |
| 62 | Chd64 | 0.75 |
| 63 | Got1 | 0.74 |
| 64 | CG42361 | 0.73 |
| 65 | aay | 0.73 |
| 66 | CG10433 | 0.73 |
| 67 | CG33229 | 0.70 |
| 68 | CG17896 | 0.70 |
| 69 | Uba2 | 0.70 |
| 70 | brat | 0.70 |
| 71 | Tcp-1zeta | 0.68 |
| 72 | BM-40-SPARC | 0.67 |
| 73 | CG7946 | 0.67 |
| 74 | CG32685 | 0.67 |
| 75 | CG10863 | 0.66 |
| 76 | nito | 0.66 |
| 77 | baf | 0.63 |
| 78 | Pino | 0.63 |
| 79 | CG3409 | 0.61 |
| 80 | Ntf-2 | 0.61 |
| 81 | CG3847 | 0.57 |
