# Supplementary material for: Genome-wide identification of neuronal activity-regulated genes in Drosophila
Source: eLife. 2016 Dec 9;5:e19942. doi: 10.7554/eLife.19942 (PMC5148613; doi:10.7554/eLife.19942)
Supplement: Figure 5—source data 3. — DOI: http://dx.doi.org/10.7554/eLife.19942.022 [file elife-19942-fig5-data3.docx]

**Figure 5 – Source Data 3.** **Overlapped ARGs in different tissue types.**

| Brains/DA only | Brains/PDF+ only | DA/PDF+ only | All |
| --- | --- | --- | --- |
| Hr38 | CG30497 | cbt | CG14186 |
| CG13055 | CG13255 | l(2)efl | CG17778 |
| CG13054 | CG33229 | CG3847 | l(1)G0148 |
| Sr | JhI-21 | aay | CG11221 |
| Grass | bnb | Pdk | Act42A |
| Ntc | BM-40-SPARC | CG7946 | baf |
| Inos |  | CG10863 |  |
| CG13868 |  |  |  |
| CG17734 |  |  |  |
| cv-c |  |  |  |
| Fmrf |  |  |  |
