# Supplementary material for: Genome-wide identification of neuronal activity-regulated genes in Drosophila
Source: eLife. 2016 Dec 9;5:e19942. doi: 10.7554/eLife.19942 (PMC5148613; doi:10.7554/eLife.19942)
Supplement: Figure 7—source data 1. — DOI: http://dx.doi.org/10.7554/eLife.19942.030 [file elife-19942-fig7-data1.docx]

**Figure 7 – Source Data 1. Motif enrichment in the 5 kb upstream regions of *ChR2-XXL-*induced ARGs.**

| **Ranking** | **Motif** | **# Genes with Binding Sites** | **Significance** |
| --- | --- | --- | --- |
| 2 | lola | 26 | 4.13E-07 |
| 86 | Eip78C | 21 | 0.000178 |
| 386 | Rel | 15 | 0.034709 |
| 418 | br | 14 | 0.066191 |
| 583 | Cf2 | 10 | 0.425856 |
