# Supplementary material for: Genome-wide identification of neuronal activity-regulated genes in Drosophila
Source: eLife. 2016 Dec 9;5:e19942. doi: 10.7554/eLife.19942 (PMC5148613; doi:10.7554/eLife.19942)
Supplement: Figure 7—source data 2. — DOI: http://dx.doi.org/10.7554/eLife.19942.031 [file elife-19942-fig7-data2.docx]

**Figure 7 – Source Data 2. Motif enrichment in the 1 kb upstream regions of *ChR2-XXL-*induced ARGs.**

| **Ranking** | **Motif** | **# Genes with Binding Sites** | **Significance** |
| --- | --- | --- | --- |
| 8 | lola | 25 | 1.55E-06 |
| 26 | Eip78C | 22 | 5.92E-05 |
| 70 | Rel | 21 | 0.000178 |
| 327 | br | 15 | 0.034709 |
| 595 | Cf2 | 9 | 0.565911 |
