# Supplementary material for: Genome-wide identification of neuronal activity-regulated genes in Drosophila
Source: eLife. 2016 Dec 9;5:e19942. doi: 10.7554/eLife.19942 (PMC5148613; doi:10.7554/eLife.19942)
Supplement: Supplementary file 1. — (A) Primers for qPCR validation. (B) Primers for reporter generation DOI: http://dx.doi.org/10.7554/eLife.19942.036 [file elife-19942-supp1.pdf]

**Table S1 Primers for qPCR validation.**

| Primer Name | Sequence |
|---|---|
| hr38 fwd pr | AACACGAGCATTAGCCACCA |
| hr38 rv pr | GGGGCATTGAGGTTCTGTCT |
| sr fwd pr | GCACTGTGCCCTTGAAACTG |
| sr rv pr | GTCGGAACGGGAGAACCTAC |
| CG14186 fwd pr | CGGACAGCATAGAGCTGGTG |
| CG14186 rv pr | GAGTCCCTGGTATTGCCTGC |
| CG30497 fwd | TATCGTTCGAGCAGGTGAGC |
| CG30497 rv | CCAGTGTGGGAAAATTGCCG |
| CG13055 fwd | CTAAGCGTTTCGCCAACACC |
| CG13055 rv | TAGGTCATCCTGCGACCTCA |
| CG17778 fwd | GCAGCATGCAGCAGACTTTT |
| CG17778 rv | CCCTTTCTCGGGCTTTTTGC |
| pk61c fwd | GATCGCGATTCGGAGGAGTT |
| pk61c rv | TTCCGGTGAAACGTACTGGG |
| cg6231 fwd | CGGAGTGGCCATCTCGTATG |
| cg6231 rv | TAAAGAAGGTGCCCAGCCAG |
| cyp4ac3 fwd | CCTTTGCCTTTGTGCCCTTC |
| cyp4ac3 rv | GCGGCTAGGAGCACCTTAAT |
| skl fwd | TGTGCAAGGTCCTGAAGCAA |
| skl rv | AATTTTTACTGCGCCTGCGG |
| CG13054 fwd | GCCCGGTCTGGATTACTACG |
| CG13054 rv | TGGTGTCTTCCTCGAAGTGC |
| grass fwd | GCTCAGTCAACTTTGCGTGG |
| grass rv | GAACTCGACCATTTTCGGCG |
| tld fwd | GGAGTGTTGCAGAGTCCCAA |
| tld rv | GGCCCAGTACGGTCTGAAAA |
| cg10514 fwd | CTCCCCATGCATCGATCTCC |
| cg10514 rv | AACTGGAAGAGCCCGTTCTG |
| cg13322 fwd | GCCGCATACCGAAGGATACA |
| cg13322 rv | AAAGTCGTCGAAGAGGCTGG |
| camta fwd | TGGAGAAGCAGAGGTTGTCG |
| camta rv | TGCGTACAGGTCTTTCCGTC |
| nmo fwd | AGCACATGACCCAAGAGGTG |
| nmo rv | CCCACCGACCATACATCCAC |
| zc3h3 fwd | CGCACTTTCTGAATCGCCAG |
| zc3h3 rv | TGGAGTCGCCAGTAGGTGTA |

**Table S2 Primers for reporters generation.**

| Primer Name | Sequence |
|---|---|
| lola reporter3 fwd | GGCCGCataagcttatctaagctcaatttgcaagctcaatttgcaagctcaatttG |
| lola reporter3 rv | GATCCaaattgagcttgcaaattgagcttgcaaattgagcttagataagcttatGC |
| rel reporter3 fwd | GGCCGCataagcttatctgggaaacccccatttgcgggaaacccccatttgcgggaaacccccatttG |
| rel reporter3 rv | GATCCaaatgggggtttcccgcaaatgggggtttcccgcaaatgggggtttcccagataagcttatGC |
| rel reporter3 fwd | GGCCGCataagcttatctgggaaacccccatttgcgggaaacccccatttgcgggaaacccccatttG |
| rel reporter3 rv | GATCCaaatgggggtttcccgcaaatgggggtttcccgcaaatgggggtttcccagataagcttatGC |
| br reporter3 fwd | GGCCGCataagcttatctcatagaccaatttgccatagaccaatttgccatagaccaatttG |
| br reporter3 rv | GATCCaaattggtctatggcaaattggtctatggcaaattggtctatgagataagcttatGC |
| cf2 reporter3 fwd | GGCCGCataagcttatctATTAGTATATATAGGTCatttgcATTAGTATATATAGGTCatttgcATTAGTATATATAGGTCatttG |
| cf2 reporter3 rv | GATCCaaatGACCTATATATACTAATgcaaatGACCTATATATACTAATgcaaatGACCTATATATACTAATagataagcttatGC |
